# Effects of a Sexual Risk-Reduction Intervention for Teenagers: A Cluster-Randomized Control Trial

**DOI:** 10.1007/s10461-022-03574-z

**Published:** 2022-01-27

**Authors:** Mayra Gómez-Lugo, Alexandra Morales, Alejandro Saavedra-Roa, Janivys Niebles-Charris, Daniella Abello-Luque, Laurent Marchal-Bertrand, Paola García-Roncallo, Eileen García-Montaño, Diana Pérez-Pedraza, Jose P. Espada, Pablo Vallejo-Medina

**Affiliations:** 1 Konrad Lorenz University Foundation, Bogotá, Colombia; 2 AITANA Research Group, Department of Health Psychology, Universidad Miguel Hernández, Avda. de la Universidad, s/n., 03202 Elche, Alicante Spain; 3 Corporación Universidad de la Costa, Barranquilla, Colombia

**Keywords:** COMPAS, Sex education, HIV, Unplanned pregnancy, Adolescence, Colombia, COMPAS, Educación sexual, VIH, Embarazo no planificado, Adolescencia, Colombia

## Abstract

This study evaluated the efficacy of the COMPAS program in the short term and 6 months after its application. For the initial sample, 2047 teenagers aged 14–19 years from 14 schools in 11 Colombian cities participated; eight schools were randomly assigned to the experimental condition and six to the control group. The participants completed self-report assessments that evaluated several variables theoretically associated with protective sexual behaviors. In the short term, the experimental group showed increased knowledge about HIV and other STIs, sexual assertiveness, self-efficacy, greater behavioral intention toward condom use, and more favorable attitudes toward HIV and condom use than the control group. After 6 months, most psychological and health variables also showed a significant positive change. In conclusion, the COMPAS program is the first school-based sexuality education program that has been shown to be effective in reducing mediating and behavioral variables associated with sexual risk reduction in Colombia.

## Resumen

Este estudio evaluó la eficacia del programa COMPAS a corto plazo y a 6 meses después de la aplicación. Para la muestra inicial participaron 2.047 adolescentes de 14 a 19 años provenientes de 12 escuelas en 11 ciudades colombianas; 8 escuelas fueron aleatoriamente asignadas a la condición experimental y 6 al grupo control. Los participantes completaron autoinformes sobre variables teóricamente asociadas con comportamientos sexuales de protección. A corto plazo, el grupo experimental mostró un mayor conocimiento sobre ITS, asertividad sexual, autoeficacia e intención conductual hacia el uso del condón y actitudes más favorables hacia el VIH que el grupo de control. Después de seis meses, la mayoría de las variables psicológicas y de salud también mostraron un cambio positivo. En conclusión, el programa COMPAS es el primer programa de educación sexual que ha demostrado ser efectivo para reducir las variables mediadoras y conductuales asociadas con la reducción del riesgo sexual en Colombia.

## Introduction

According to the latest UNAIDS report [1], at least 37.7 million people around the world are living with HIV. Adolescents and young people represent a growing share of people living with HIV worldwide. According to UNICEF, in 2020, approximately 1.75 million adolescents between the ages of 10 and 19 were living with HIV worldwide, and in that year alone, 150,000 adolescents globally were newly infected with HIV; if current trends continue, there will be approximately 183,000 annual new HIV infections among adolescents in 2030 [2]. Colombia ranks third in Latin America, after Brazil and Mexico, in terms of new cases of HIV [3]. Likewise, data from the UNAIDS [4] indicate that there are approximately 3100 teenagers living with HIV in the country. Colombia had the highest rates of infection in these age groups in Latin America in 2018.

On the other hand, approximately 16 million teenagers between the ages of 15 and 19 years and two million girls under the age of 15 become pregnant worldwide each year; teenage pregnancy rates in Latin America and the Caribbean continue to be the second highest globally, surpassed only by Sub-Saharan Africa. Maternal mortality is one of the leading causes of death among teenagers and young people aged 15 to 24 years in the Region of the Americas [5]. In Colombia, Profamilia and the Plan International Foundation, led by the Ministry of Health, found that 13.8% of teenage girls between the ages of 13 and 19 years have been or currently were pregnant, with the highest levels of teenage pregnancy being in rural areas of the country (18.6%). Recent studies have shown that, without sex education programs, Colombian teenagers are at high risk of unplanned pregnancies [6] or acquiring sexually transmitted infections (STIs), mainly due to inconsistent condom use [7].

Various prevention programs have been developed from different theoretical and methodological perspectives to reduce the rates of unplanned pregnancies, HIV, and other STIs [8–10]. However, the conclusions provided by meta-analyses of HIV prevention interventions (comparing the results reported by various controlled studies), show that the most effective programs usually have the following characteristics [11, 12]: (1) are based on behavior modification models, such as the information-motivation-skills model (IMB), social learning theory (SLT) [13–15], social cognitive theory [16], the theory of reasoned action [17], and the theory of planned action [18]; (2) have a duration of four hours or more; and (3) contain attitudinal components, educational information, and behavioral skills training. The least effective programs were those that attempted to induce HIV-related fear. Highly effective programs tend to increase sexual health knowledge; promote favorable attitudes toward HIV and the use of protective methods; increase self-efficacy to use condoms; increase behavioral intention, including intention to use condoms and intention to refuse sex; and increase condom use among adolescents [19].

The competencies for adolescents with a healthy sexuality (COMPAS) program was developed for Spanish adolescents based on these criteria and the theoretical models of behavior modification, specifically the social learning theory model [15] and the Information-Motivation-Behavioral Skills Model [13]. The program has a duration of five hours and targets attitudinal components. In a Spanish sample, the COMPAS program has been experimentally demonstrated to contribute to the reduction of risky sexual behaviors and enhance protective behaviors against the transmission of HIV, other sexually transmitted diseases (STDs), and unplanned pregnancies among teenagers and young people in the short term and 24 months after its application [20, 21]. In addition, through several controlled studies [22, 23], the program has also been shown to be effective in increasing knowledge of HIV and other STDs, risk perception of unprotected sex, condom self-efficacy, and promoting positive attitudes toward HIV and sexuality. It also promotes favorable attitudes toward condom use and HIV testing, thus reducing HIV phobia. On the other hand, while the COMPAS program seems to have the right components to reduce risky sexual behavior, and although it has been culturally adapted for use in Colombia [24] and this version has theoretically demonstrated suitability for addressing sexuality education for teenagers aged 13–19 years, it has not yet been demonstrated to be effective in reducing risky sexual behavior [25]. Therefore, this study aimed to evaluate the effectiveness of the Colombian version of the COMPAS program immediately after its implementation and after 6 months in a sample of teenagers from different cities in Colombia.

## Methods

### Study Design and Participants

**Fig. 1:**
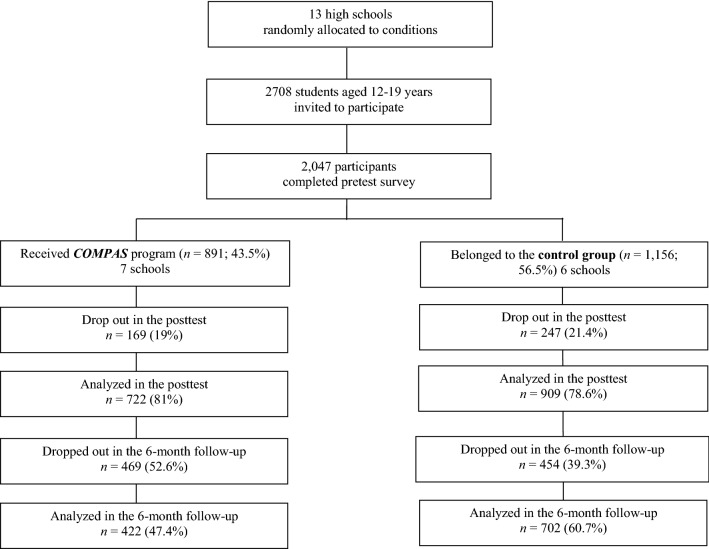
Progress of participating students though the trial

In the present cluster-randomized trial, 2047 teenagers aged 12–19 years initially participated. The participants were from 11 cities in central and coastal Colombia, corresponding to the urban and rural areas of the country. All the participants were authorized by their parents and/or guardians, who signed an informed consent form, to participate in the study. Only students between the 8th and 11th grades of high school participated. The participants were required not to have previously participated in formal sex education programs. The study excluded the participants who did not provide consent and those whose legal guardians did not provide consent. The exclusion also applied to those who, despite attending the evaluation phases, did not participate in the group sessions. The sociodemographic characteristics of the participants in each group are described in Table 1, and Fig. 1 shows data on the sample recruitment process and the percentage of dropouts at each stage of the project.

**Table 1:** Sociodemographic characteristics and self-reported behaviors of baseline participating students by intervention condition

Characteristics	*COMPAS* program (*n* = 891)	Control group (*n* = 1156)	Total (*N* = 2047)	Test statistics^a^	*p* value	Cohen’s *d*
No. (*%*) female	491 (55.25)	575 (49.8)	1,066 (52.1)	7.87	0.02	0.10
Mean age (*SD*), years	15.48 (1.36)	15.05 (1.30)	15.24 (1.35)	− 7.10	< .001	0.32
No. (*%*) who have married parents	263 (30.3)	425 (37.6)	688 (34.4)	11.83	0.001	0.15
No. (%) Socioeconomic level						
0	7 (0.8)	5 (0.5)	12 (0.6)	152.24	< 0.001	0.12
1	300 (35.7)	191 (17.6)	491 (25.5)			
2	183 (21.8)	423 (39)	606 (31.5)			
3	224 (26.7)	377 (34.7)	601 (31.2)			
4	91 (10.8)	52 (4.8)	143 (7.4)			
5	31 (3.7)	19 (1.8)	50 (2.6)			
6	4 (0.5)	18 (1.7)	22 (1.1)			
Religious practices (yes)						
Daily	36 (4.1)	57 (5)	93 (4.6)	7.32	0.29	-
At least once a week	234 (26.5)	314 (27.7)	548 (27.2)			
At least once every 2 weeks	79 (9)	102 (9)	181 (9)			
At least once every three weeks	41 (4.6)	71 (6.3)	112 (5.6)			
At least once a month	183 (20.7)	246 (21.7)	429 (21.3)			
At least once a year	186 (21.1)	200 (17.6)	386 (19.1)			
Never	123 (13.9)	145 (12.8)	268 (13.3)			
No. (*%*) Sexual orientation						
No socio-sexual contacts or reactions	20 (2.4)	33 (3.1)	53 (2.8)	9.36	0.22	-
Exclusively heterosexual	776 (91.5)	930 (88.4)	1,706 (89.9)			
Predominantly heterosexual, only incidentally homosexual	20 (2.4)	40 (3.8)	60 (3.2)			
Predominantly heterosexual, but more than incidentally homosexual	5 (0.6)	12 (1.1)	17 (0.9)			
Equally heterosexual and homosexual	13 (1.5)	20 (1.9)	33 (1.7)			
Predominantly homosexual, but more than incidentally heterosexual	1 (0.1)	5 (0.5)	6 (0.3)			
Predominantly homosexual, only incidentally heterosexual	2 (0.2)	3 (0.3)	5 (0.3)			
Exclusively homosexual	11 (1.3)	9 (0.9)	20 (1.1)			
No. (*%*) Sexually experienced	475 (56.4)	572 (52.2)	1,047 (54)	2.42	0.06	-
No. (*SD*) Children	0.02 (.21)	0.03 (.30)	0.03 (0.27)	0.96	0.33	-
Percentage of condom use (0–100) (*SD*)	57.29 (32.58)	61.28 (31.01)	59.33 (31.82)	1.47	0.14	-
No. (*%*) Consistent condom use	46 (18.5)	53 (19.5)	99 (19)	0.08	0.76	-

No. = valid frequency. The socioeconomic levels are listed according to the Colombian official strata divisions

^a^*T*-test for continuous variables and χ^2^ test for categorical variables

### Intervention

#### COMPAS Program (Competencies for Adolescents with a Healthy Sexuality)

The COMPAS program is framed within the SLT [26] and the information-motivation-behavioral (IMB) skills model for AIDS-preventive behavior [13]. It is a program originated in Spain, designed to reduce sexual risk behaviors in teenagers, promote sexual health, improve decision-making skills, and promote sexual assertiveness. In the version adapted for use in Colombia, the program included role-playing exercises adapted to the experiences of Colombian adolescents and activities adapted to socio-economic conditions and the vulnerabilities of the Colombian youth, specifically those associated with sexual risk behaviors, such as limited access to contraception, STI testing, and abortion). Moreover, the research included a sexual diversity component, considering that the original version only addressed heteronormative relationships [24]. The COMPAS program applies a participatory action methodology using cognitive-behavioral intervention activities, such as role-playing, brainstorming, gamified experiences, cognitive restructuring, training in social skills, problem-solving, self-instructions, and decision-making, among others. These activities are intended to modify knowledge mainly about HIV and other STDs, and improve attitudes toward HIV protection behaviors, self-efficacy, and sexual assertiveness [27]. These components are important for the modification of sexual behavior [28].

### Procedure

The project was submitted to the ethics and scientific committee of the participating universities, and upon obtaining approval, the search began for educational institutions willing to participate voluntarily. Subsequently, informed consent was requested from the legal guardians of the underage participants, as well as written consent from the teenagers. The schools were selected to meet similar conditions in terms of in-person attendance, mixed-gender education, public or private institution, and the socioeconomic status of the institution. The schools were randomly assigned to either the control or the experimental group.

Both the experimental and control groups were administered a list of evaluative questionnaires as a group in the first week. To preserve confidentiality, each participant was assigned an identification code to link the data of each participant in the different phases of the process. Only the person responsible for the study had correspondence information between the code and each participant’s data. The experimental group participants received the program, which involved one group session per week, with 25–30 participants. The experimental group participants received the program in person at their educational institution. The sessions were held during class hours, varying the schedule so that the students did not experience conflicts with other classes. Each group had a single researcher throughout the program who facilitated five intervention sessions of one hour once per week.

Participation in each of the sessions was voluntary; however, 85.4% of the experimental group participants attended all five sessions. The control group participants received no intervention during the 5 weeks of intervention in the experimental group. Once this time period was completed, the participants completed a posttest and a follow-up test. Once the research process was completed, a five-session intervention was carried out for those who wanted to participate, adhering to the ethical principles of research with human participants.

The program implementers had to (a) have a minimum degree in psychology; and (b) have received training in sexual and reproductive health, specifically in the application of the COMPAS program. This training was complemented by discussion after each session to resolve possible doubts and discuss them with the project coordinator. As the program is highly structured, the implementers had to strictly follow the application manual in all sessions.

After the initial evaluation week, the control group participants had a period of 5 weeks in which they did not receive any treatment. In week 7, the posttest was applied; the follow-up test was applied 6 months later. Subsequently, the COMPAS program was applied to the control group (following the principle of beneficence). The participants did not receive any reward for their participation in this study.

### Measures

This study proposes a comprehensive view to evaluate the effectiveness of the COMPAS program. To this effect, we have included socio-demographic and behavioural measures, as well as safe sex predictors.

#### Sociodemographic Variables

The sociodemographic characteristics of the participants were evaluated using a semi-structured ad hoc questionnaire. Educational level, age, gender, sexual experience (initiation of sexual intercourse), religion and frequency of religious practice, sexual orientation using the Kinsey scale (It consists of eighth categories: from *No socio-sexual contacts or reactions* to *Exclusively homosexual*), and family type (parents separated or living together) were evaluated. The socioeconomic characteristics are listed in Table 1, according to the Colombian Official strata divisions. The national classification identifies groups with similar socioeconomic characteristics, where zero equals extreme poverty, and six represents a high economic income.

#### Behavioral Measures

Sexual behaviors were assessed using the following components: age of sexual debut (vaginal, anal, and oral), number of people with whom they had had sexual intercourse in their lifetime (sexual experienced), percentage of condom use (rated on a scale ranging from 0 to 100%), consistent condom use (yes/no), and condom use at last vaginal, anal, and oral penetration (yes/no)*.*

#### Safe-Sex Predictor Measures

The safe-sex predictors measured include HIV knowledge, attitudes towards HIV/ITS infection, perceived self-efficacy, sexual assertiveness, perceived norms related to peer’s condom use, and condom use intention.

##### HIV and STD Knowledge (ECI)

The Colombian version of the HIV and STD knowledge scale was used [29, 30]. It consists of five factors that assess general knowledge about HIV, condom use, means of transmission of STDs, and knowledge about other STIs. This scale is composed of 24 items divided into the five factors described above. The response scale is *True*, *False*, or *Don’t know*. The internal consistency of the Colombian version was 0.87 in this study.

##### HIV-Related Attitudes (HIV-AS)

Attitudes toward HIV are defined as the tendencies to behave toward the following: barriers to safer sex, HIV and STD testing, condom use, and attitudes toward people living with HIV. These types of attitudes were assessed using the HIV-AS questionnaire, originally designed by Espada et al. [31] and validated for use in Colombia by Gómez-Lugo et al. [32]. This scale is composed of 12 items with four Likert-type response options ranging from 1 (*strongly disagree*) to 4 (*strongly agree*). The internal consistency of the questionnaire ranged from 0.56 to 0.73 in the present study.

##### Sexual Assertiveness

This type of assertiveness is made up of three main components: *sexual assertiveness of initiation*, defined as the ability to request the initiation of sexual intercourse when desired; *sexual assertiveness of refusal*, defined as the ability to refuse sexual intercourse when not desired; and *sexual assertiveness of negotiation for the avoidance of pregnancy-sexually transmitted diseases/infections*, which is conceptualized as the ability to negotiate the use of protection and contraception when there is a risk of unplanned pregnancy or STIs. In the present study, the version validated for use in Colombia was used [33]. The scale is composed of nine items rated on a Likert-type response scale with values ranging from 0 to 4 points. The instrument has been shown to be valid and reliable in both men and women, obtaining an alpha coefficient ranging from 0.61 to 0.89.

##### Self-Efficacy

Consistent and correct condom use is the only method that prevents unintended pregnancy and STIs [34]. However, low self-efficacy for its use considerably decreases its effectiveness [35]; hence, it is important to assess its effectiveness [36]. Two scales were used to measure this type of self-efficacy. The first was the Condom Use Errors/Problems Survey (CUES) in its validated version for use in Colombia with a seven-point Likert-type response scale, which evaluates the perception of self-efficacy for the use of condoms in future sexual relations. The first item evaluates the perceived likelihood of using condoms correctly in future sexual relations (checking the expiration date, opening the package, inserting and unrolling it before having intimate contact, etc.). The second item evaluates the perception of how easy/difficult the use of condoms is during penetrative intercourse. The third item assesses perceived confidence in the ability to put on a condom correctly during vaginal, anal, and/or oral intercourse.

##### Behavioral Intention

This component was evaluated through two factors that refer to the intention to engage in healthy sexual behaviors during the next 2 months. The first factor is composed of three items that assess the probability of seeking, using, and requesting the use of condoms before initiating penetrative sexual intercourse. This item had a reliability coefficient of α = 0.80. The second factor is composed of two items that assess the intention to have sex under the influence of drugs (alcohol and psychoactive substances). This item had a reliability coefficient of α = 0.71.

##### Normative Perception

This variable refers to the participants’ perception of condom use by their peers. The construct was assessed by two items from a semi-structured ad hoc questionnaire. The first item was “Do you think peers your age use condoms in their sexual relationships?” (*yes/no*). The second item related to the perceived frequency of condom use: “How often do you think your peers use condoms during sex?” This was assessed using a four-point Likert-type response scale ranging from *Always* [4] to *Never* [1].

### Statistical Analysis

The characteristics of the sample were analyzed using descriptive statistics. Baseline differences between the experimental and control groups were analyzed using the *t*-test for quantitative variables (numerical) and cross-tables (χ^2^) for categorical variables. An intent-to-treat approach was used; therefore, data from all participants were analyzed, regardless of the level of intervention received [37]. The attrition rate in the posttest and the 6-month follow-up was calculated. Differences in sociodemographic variables and main outcomes (e.g., condom use) between participants who dropped out and those who were retained were also investigated using *t-*tests and cross-tables. When differences were statistically significant, Cohen’s *d* effect size was estimated. The coefficients were interpreted as follows: 0.20 = small, 0.50 = medium, and 0.80 = large (Cohen, 1988). Following previous studies (e.g., [21]), intervention effects compared to the control group were analyzed using generalized estimating equations (GEE). Values were adjusted for baseline differences, participants’ age, gender, and socioeconomic level. The GEE models are valuable for evaluating interventions in cluster-randomized control trials because they control for correlations among responses when the sample is clustered within schools [38]. The unit of randomization was the school, while the individual was the unit of analysis. Consequently, the school variable was controlled for in all the analyses. All analyses were performed using IBM SPSS Statistics for Windows, version 26.

## Results

### Attrition

Figure 1 shows the flow of participants throughout the study. In the posttest, there was no statistically significant difference in the loss of participants between the two experimental conditions (χ^2^ = 1.78, *p* = 0.18). However, teenagers who dropped out were older (*p* < 0.001; *d* = 0.44), and a higher proportion were sexually experienced (χ^2^ = 13.84, *p* < 0.001; OR = 0.65[.52, 0.81]) and women (χ^2^ = 6.35, *p* < 0.01; *OR* = 0.75 (0.60, 0.94) than those who remained in it. Consistent condom use and the percentage of condom use were unrelated to the loss of participants.

At the 6-month follow-up, there were significant differences between the experimental conditions (χ^2^ = 36.29, *p* < 0.001; OR = 0.58 [0.48, 0.69]). A higher proportion of participants in the COMPAS program condition dropped out compared to the control group. Teenagers who dropped out during follow-up were older (*p* < 0.001; *d* = 0.51), and a higher proportion were sexually experienced (χ^2^ = 19.48, *p* < 0.001; *OR* = 0.66 (.55, 0.79)) than those who remained in it. The participants’ gender, consistent condom use, and percentage of condom use were unrelated to the loss of participants in the follow-up.

### Participants’ Characteristics

The mean age was 15.24 years (SD= 1.37; *range* = 12–19 years), with a significant difference in this variable between the groups, although with a small effect size (*p* < 0*.*001; d=0.32); the experimental group (EG) had a higher mean age. A total of 52.1% of the participants were women, and their parents were married in 34.4% of the cases. Socioeconomic level was also a contrasting variable between the groups, with significant differences (*p*<0*.*001; d=0.12); most of the participants belonged to stratum 2 and 3, indicating a prevalence of medium-small purchasing power in both groups.

Regarding the psychosexual characteristics of the adolescents, 89% of the sample identified themselves as exclusively heterosexual, and only 0.27% of the participants had children. Regarding sexual characteristics, 54% of the adolescents were sexually active and used condoms 31% of the time, with only 19% of the sample consistently using condoms. The age of initiation of sexual intercourse was evaluated, finding that the mean age was 14.43 years old (SD=1.56) for vaginal penetration, 14.36 years old (SD=1.73) for anal penetration, 14.35 years old (SD=1.89) for masturbation, 14.34 years old (SD=1.79) for oral sex, and 13.28 years old (SD=1.8) for *dry humping* (defined as consensual genital stimulation through clothing). Table 1 summarizes the characteristics of the participants by group at the beginning of the study.

### Outcome Measures

#### Short-Term Program Effects

Table 2 shows the changes in the marginal means of the variables at baseline, post-test, and 6-month follow-up. Table 3 shows the changes observed at posttest and follow-up with respect to the baseline (respectively).

**Table 2:** Estimated marginal means of the outcomes between pre-, post- and 6 months follow up by experimental condition

	COMPAS program			Control group		
	Pre-treatment mean (95% CI) or *N* (%)	Post-treatment mean (95% CI) or *N* (%)	6-month follow-up mean (95% CI) or *N* (%)	Pre-treatment mean (95% CI) or *N* (%)	Post-treatment mean (95% CI) or *N* (%)	6-month follow-up mean (95% CI) or *N* (%)
*Precursors*						
HIV and STIs knowledge, *M*						
General HIV (0–8)ª	4.47 (4.43, 4.51)	5.33 (5.19, 5.48)	5.19 (5.01, 5.37)	4.43 (4.40, 4.47)	4.31 (4.18, 4.44)	4.21 (4.03, 4.38)
Condom use (0–2)ª	1.10 (1.08, 1.12)	1.36 (1.29, 1.42)	1.21 (1.13, 1.29)	1.11 (1.09, 1.13)	1.02 (0.96, 1.07)	0.97 (0.91, 1.04)
Routes of transmission (0–6)ª	2.13 (2.09, 2.16)	3.10 (2.97, 3.24)	3.01 (2.84, 3.18)	2.10 (2.07, 2.13)	1.90 (1.80, 1.99)	1.73 (1.61, 1.85)
Prevention (0–3)ª	1.06 (1.04, 1.08)	1.57 (1.49, 1.66)	1.57 (1.45, 1.68)	1.01 (00.99, 1.03)	0.96 (0.90, 1.02)	1.05 (0.97, 1.12)
Other sexual infections (0–6)ª	1.15 (1.11, 1.18)	1.87 (1.73, 2.01)	1.77 (1.60, 1.95)	1.11 (1.08, 1.14)	.99 (.90, 1.07)	.91 (.80, 1.02)
Total (0–24)ª	9.92 (9.84, 10)	13.30 (12.89, 13.72)	12.71 (12.18, 13.24)	9.80 (9.73, 9.87)	9.20 (8.92, 9.49)	8.81 (8.43, 9.19)
HIV-related attitudes, *M*						
Obstacles (3–12)ª	9.19 (9.14, 9.24)	9.71 (9.57, 9.84)	9.23 (9, 9.45)	9.14 (9.10, 9.19)	9.12 (8.98, 9.25)	9.14 (8.97, 9.31)
HIV test (2–8)ª	6.85 (6.81, 6.89)	6.97 (6.87, 7.07)	6.69 (6.55, 6.83)	6.79 (6.75, 6.82)	6.54 (6.45, 6.64)	6.36 (6.23, 6.48)
Condom (4–16)ª	12.67 (12.62, 12.73)	13.52 (13.35, 13.68)	13.11 (12.87, 13.36)	12.62 (12.57, 12.67)	12.64 (12.48, 12.81)	12.61 (12.41, 12.80)
People living with AIDS (3–12)ª	3.92 (3.88, 3.96)	3.73 (3.61, 3.85)	3.69 (3.52, 3.86)	3.90 (3.87, 3.94)	3.98 (3.88, 4.08)	4.13 (4, 4.26)
Total (12–48)ª	32.69 (32.58, 32.80)	34.06 (33.71, 34.40)	32.82 (32.32, 33.92)	32.55 (32.45, 32.65)	32.33 (31.99, 32.67)	32.22 (31.76, 32.68)
Self-efficacy (1–7)ª, *M*	5.07 (5.04, 5.11)	5.44 (5.35, 5.54)	5.36 (5.24, 5.49)	5.08 (5.05, 5.11)	5.05 (4.97, 5.14)	4.99 (4.90, 5.09)
Behavioral intention (5–25)ª, *M*	20.94 (20.86, 21.03)	21.53 (21.28, 21.79)	21.01 (20.66, 21.36)	20.85 (20.77, 20.93)	20.88 (20.67, 21.10)	20.59 (20.32, 20.87)
Normative perception, *N* (%)						
Peers use condom (yes), *N* (%)	499 (60.1)	417 (60.9)	228 (58.3)	630 (57.1)	480 (56.7)	371 (57)
Peers use always or almost always condom, *N* (%)	348 (40.2)	254 (37.4)	133 (34.1)	371 (33.7)	274 (18)	211 (20.3)
Sexual Assertiveness, *M*						
Initiation (3–15)ª	6.18 (6.12, 6.24)	7.55 (7.25, 7.85)	7.49 (7.07, 7.91)	6.26 (6.20, 6.32)	6.44 (6.17, 6.70)	6.98 (6.63, 7.34)
No shyness/refusal (3–15)ª	8.11 (8.01, 8.229	8.82 (8.48, 9.16)	9.07 (8.50, 9.63)	7.95 (7.85, 8.06)	8.18 (7.81, 8.55)	8.21 (7.74, 8.68)
STIs (3–15)ª	10.18 (10.04, 10.31)	10.63 (10.30, 10.96)	22.24 (21.23, 23.24)	9.95 (9.81, 10.08)	9.53 (9.16, 9.90)	22.89 (21.84, 23.94)
*Sexual behavior*						
Age of first vaginal penetration, *M*	14.43 (14.39, 14.46)	14.50 (14.32, 14.68)	14.43 (14.03, 14.83)	14.44 (14.41, 14.47)	14.40 (14.27, 14.54)	14.37 (14.15, 14.58)
Age of first anal penetration, *M*	14.36 (14.28,14.43)	14.55 (14.23, 14.87)	14.77 (14.03, 15.52)	14.30 (14.24, 14.36)	14.34 (14.13, 14.54)	13.55 (12.16, 14.94)
Age of first oral sex, *M*	14.28 (14.23, 14.34)	14.63 (14.38, 14.88)	14.28 (13.88, 14.69)	14.27 (14.23, 14.30)	14.34 (14.19, 14.49)	14.05 (13.52, 14.59)
Percentage of condom use (0–100), *M*	60.76 (59, 61.90)	67.86 (63.57, 72.14)	68.28 (61.54, 75.02)	61.13 (60.09, 62.18)	61.23 (57.50, 64.96)	63.48 (57.97, 69)
Consistent condom use (yes), *N* (%)	46 (18.5)	42 (17.4)	26 (19.3)	53 (19.5)	36 (15.6)	25 (13.8)
Condom use last vaginal, penetration (yes), *N* (%)	170 (51.8)	147 (66.8)	93 (69.9)	190 (59.4)	156 (68.1)	112 (64.4)
Condom use last anal penetration (yes), *N* (%)	31 (16.3)	43 (41.3)	24 (39.3)	44 (28.9)	32 (32.7)	34 (31.4)
Condom use last oral sex episode (yes), *N* (%)	46 (8.5)	33 (19.3)	21 (20.4)	39 (14.6)	23 (12.1)	22 (13.3)
Sexual partners in a lifetime, *M*	1.49 (1.48, 1.51)	1.70 (1.51, 1.89)	1.88 (1.52, 2.23)	1.51 (1.50, 1.52)	1.56 (1.40, 1.73)	1.93 (1.68, 2.17)

*M* = Mean (for continuous variables) and *N* (%) for categorical variables was reported

ªRange of possible responses to the outcomes

#### Safe-Sex Predictor Measures

In the posttest, there were statistically significant changes in 15 of the 17 safe-sex predictor variables that were analyzed. The GEE analysis revealed a positive impact of the COMPAS program on knowledge of HIV and other STDs, HIV-related attitudes, self-efficacy, behavioral intention, and sexual assertiveness compared to the control group.

#### HIV and STDs Knowledge

Post-hoc analyses showed an increase in post-treatment response means, including the total score (AOR = 29.33; 95% CI 18.97–45.34; *p*< 0.001). There were significant effects on the HIV knowledge variable and all its subscales. These significant increases in measures were not evident in the control group, indicating short-term improvement in HIV and STD knowledge after the application of the COMPAS program.

#### HIV-Related Attitudes

With respect to attitudes toward HIV, there was a significant positive effect on the EG (AOR = 3.91; 95% CI 2.68–5.70; *p* < 0.001). The comparative analyses (see Table 3) show differences in all the variables in the short-term measures, with the variable of attitudes toward condom use showing the greatest change (AOR = 2.33; 95% CI 1.94–2.80; *p*< 0.001). Regarding the CG, it can be observed in Table 2 that the variables remained constant in all measures, except for attitudes toward people living with HIV, for which an increase in the means was observed, indicating worse attitudes toward people living with HIV. The results indicate an improvement in attitudes toward HIV in the short term after the implementation of the program.

**Table 3:** Generalized linear models and effect size estimates for the intervention effect (compared to the control group) on precursors to sexual behavior in the posttest and 6-month follow-up

Outcomes	Post-treatment		6-month-follow-up	
	AOR (95% CI)	*p* value	AOR (95% CI)	*p* value
HIV and STIs knowledge				
General HIV	2.37 (2.02, 2.79)	< .001	2.05 (1.69, 2.50)	< 0.001
Condom use	1.29 (1.20, 1.39)	< .001	1.11 (1.01, 1.21)	0.01
Routes of transmission	2.65 (2.29, 3.06)	< .001	2.41(2.01, 2.87)	< 0.001
Prevention	1.66 (1.52, 1.81)	< .001	1.65 (1.46, 1.85)	< 0.001
Other sexual infections	2.06 (1.77, 2.39)	< .001	1.87 (1.55, 2.25)	< 0.001
Total	29.33 (18.97, 45.34)	< .001	16.23 (9.39, 28.05)	< 0.001
HIV-related attitudes				
Obstacles	1.68 (1.44, 1.96)	< .001	1.04 (0.82, 1.31)	0.74
HIV test	1.12 (1.01, 1.26)	.04	0.85 (0.73, 0.99)	0.03
Condom	2.33 (1.94, 2.80)	< .001	1.55 (1.19, 2.01)	0.007
People living with AIDS	0.82 (0.71, 0.94)	.006	0.79 (0.66, 0.95)	0.01
Total	3.91 (2.68, 5.70)	< .001	1.13 (0.67, 1.92)	0.63
Self-efficacy	1.44 (1.30, 1.61)	< .001	1.33 (1.17, 1.52)	< 0.001
Behavioral intention	1.80 (1.35, 2.40)	< .001	1.07 (0.73, 1.57)	0.71
Normative perception				
Peers use condom	0.97 (0.93, 1.02)	.31	0.96 (0.91, 1.01)	0.17
Peers use always or almost always condom	0.96 (0.92, 1)	.09	0.95 (0.90, 1.01)	0.14
Sexual assertiveness				
Initiation	3.93 (2.87, 5.39)	< .001	3.70 (2.39, 5.73)	< 0.001
No shyness/refusal	2.02 (1.39, 2.94)	< .001	2.60 (1.42, 4.72)	0.002
STIs	1.57 (1.09, 2.26)	.01	12.06 (0.53, 11.01)	< 0.001

Each analysis was adjusted for the baseline measure, gender, age, socioeconomic-level (*estrato*) and school-level

*AOR* adjusted odds ratio, *CI* confidence interval

#### Self-Efficacy

Self-efficacy for condom use also showed a significant positive difference at post-treatment among the EG (AOR = 1.44; 95% CI 1.30–1.61; *p*< 0.001). In Table 2, we can observe that among the CG, the means of self-efficacy decreased, which indicates a decline in this construct.

#### Behavioral Intention

Behavioral intention, understood as the probability of engaging in condom-seeking, condom-using, and condom-requesting behaviors before initiating penetrative intercourse, was shown to be modified by the implementation of the COMPAS program (AOR = 1.80; 95% CI 1.35–2.40; *p* < 0.001). In the CG, this type of intention remained constant at post-treatment. In contrast, in the EG, the means increased at post-treatment.

#### Normative Perception

The perception of condom use by peers expressed by teenagers in the EG was the only variable that did not show any significant change in either of its two factors: perception of condom use and perception of consistent condom use by peers.

#### Sexual Assertiveness

According to the post-hoc analyses, sexual assertiveness was a component positively affected in the short term by the intervention, with significant differences in the factors of assertiveness of initiation, refusal, and negotiation of the use of protective practices against STDs and unplanned pregnancies among the EG (*p* < 0.01).

### Six-Month Program Effects

In this phase, the effect of the intervention continued to show a change in 13 of the 17 variables analyzed, two fewer than in the posttest. A decrease was found in behavioral intention to use condoms and in attitudes toward HIV, specifically those related to HIV-protective behaviors when faced with obstacles. However, most psychological and behavioral variables showed significantly positive changes from baseline measures.

#### Safe-Sex Predictor Measures Outcomes

Knowledge of HIV in the EG was the construct with the greatest stability in the measures at the 6-month follow-up phase, and all the differences in the subscales and the total scores of the measures (AOR = 16.23; 95% CI 9.39–28.05; *p* < 0.001) were significantly positive. These significant differences contrast with what was found in the CG (see Table 2) in which most of the means decreased at follow-up. Regarding attitudes toward HIV, it was found that two of the three variables in which significant differences had been found remained stable: attitudes toward condom use and attitudes toward people living with HIV (*p* = 0.63). Regarding self-efficacy for condom use, we also found significantly positive differences at follow-up, in contrast to the decrease observed in the means of the CG at follow-up. On the other hand, the perception of condom use by peers, as in the short-term follow-up, showed no significant change from the baseline. Regarding sexual assertiveness, it was observed that the three measures of initiation (AOR = 3.70; 95% CI 2.39–5.73; *p* < 0.001), rejection (AOR = 2.60; 95% CI 1.42–4.72; *p* < 0.002), and negotiation of condom use (AOR = 12.06; 95% CI 0.53–11.01; *p* < 0.001) continued to show positive significant differences with respect to the posttest. This indicates a long-term change in most of the measures due to the application of the COMPAS program.

#### Behavioral Outcomes

Teenagers in the EG showed higher rates of healthy sexual behaviors, thus finding significantly positive differences in the percentage of condom use during penetrative sexual intercourse (AOR = 7.52; 95% CI 0.41–16.62; *p* <0.05), which could indicate an increase in self-efficacy following the application of the COMPAS program (see Table 4). On the other hand, significant differences were also found in the EG in the number of lifetime sexual partners (AOR = 7.52; 95% CI 0.41–16.62; *p* <0.05), with an increase in the mean at follow-up; however, this increase was also evident in the CG. Regarding the variables of consistent condom use and condom use during anal and oral sex, no significant differences were found with respect to the baseline; however, there was a slight increase in the means of the responses (see Table 2).

**Table 4:** Generalized linear models and effect size estimates for the intervention effect (compared to the control group) on sexual behavior outcomes in 6 months follow-up for sexually experienced adolescents (*n* = 1047)

Outcomes	6-month-follow-up	
	AOR (95% CI)	*p* value
Percentage of condom use (0–100)	7.52 (0.41, 16.62)	0.03
Consistent condom use (Yes/No)	0.93 (0.83, 1.03)	0.19
Condom use last vaginal penetration (Yes/No)	1.12 (1.01, 1.25)	0.02
Condom use last anal penetration (Yes/No)	1.09 (0.88, 1.35)	0.40
Condom use last oral sex episode (Yes/No)	1.08 (0.96, 1.21)	0.19
Sexual partners in a lifetime (number)	1.46 (1.02, 2.08)	0.03

Each analyses were adjusted for the baseline measure, gender, age, socioeconomic-level and school-level

*AOR* adjusted odds ratio, *CI* confidence interval

## Discussion

The objective of this cluster-randomized trial was to evaluate the efficacy of the Colombian version of the COMPAS program in a sample of Colombian teenagers. The results indicate that the program is effective in the short term in increasing the variables theoretically related to sexual risk behaviors, namely general knowledge about HIV and other STDs, attitudes of protection against HIV, self-efficacy, and sexual assertiveness, compared to the control group. These results are in accordance with the hypotheses initially proposed and with the findings of previous studies in which the short-term efficacy of the COMPAS has been demonstrated in Spanish samples [20, 23, 39, 40].

Regarding efficacy at the 6-month follow-up, the measures remained constant from the post-treatment phase to the follow-up phase in most of the theoretically related variables, namely knowledge about HIV and other STDs, attitudes toward condom use, and sexual assertiveness, which is indicative of the medium-term efficacy of the COMPAS. This is in line with expectations and findings from previous studies that have tested efficacy after 12 and 24 months of implementation [21, 41]. However, the data indicate a decrease in behavioral intention to use condoms at follow-up and in attitudes toward condom use when barriers are present. This is related to the results of Albarracín et al. [11], who found that sexual education was applied with some regularity in teenagers to maintain changes in psychological and behavioral variables in the long term.

With the increase in these precursor variables, the intervention would be expected to influence sexual behavior in accordance with theoretical models of health [42–44] and previous empirical studies [45, 46]. This change was evidenced in the follow-up measures, revealing significant differences in the percentage of condom use and condom use during the last vaginal penetrative intercourse, with the CG being the group with higher scores in both the posttest and follow-up. However, in the follow-up phase, no significant differences were obtained between the EG and the CG regarding the percentage of condom use during sexual practices, such as anal and oral sex; this is in accordance with the study by Vallejo-Medina et al. [25], who through an analysis of the co-occurrence of the words and contents of the Colombian version of the COMPAS program, found the centralization of prevention strategies in vaginal practices, limiting others related to risk reduction in oral and anal sexual practices.

The Colombian version of the COMPAS failed to change the normative perception of condom use by peers, in contrast to the Spanish version [41]. This difference may be explained by the abrupt differences in normative perceptions found between the Spanish and Colombian samples from the baseline. In the Spanish sample, about 88% of the participants perceived that their peers used condoms, while in the Colombian sample, only 60% perceived condom use by their peers, and only 40% perceived condom use to be consistent [47]. This radical difference in normative perception may be due to the following reasons: (1) the differences in the rates of adolescents who gave birth, which was 128,665 [48] in Colombia in 2018, compared to 1167 in Spain [48, 49]; (2) the differences in the proportions of children under 15 living with HIV in each country (in Colombia the figure was approximately 3600, while in Spain the proportion was less than 100 children); and (3) the fact that most of the participants’ peers and friends did not attend the intervention, so no changes in normative perception would be expected [50].

### Limitations of the Study

Despite the good indicators shown by this research, this study is not free of limitations, which must be considered when interpreting the results. First, although the program was applied in 11 different cities, Colombia is a country with extensive multiculturalism and high rates of economic, educational [51], and educational inequalities [52], rendering some populations more vulnerable to events such as STDs, sexual abuse [53, 54], and unplanned pregnancies [55]. Therefore, the results of this study cannot be generalized to all teenagers in the country. Another limitation was the differences in some sociodemographic variables between the EG and CG, specifically the mean age, which was higher in the EG, and the socioeconomic level (stratum), which was higher in the CG. Furthermore, the teenagers who participated in the study were mostly heterosexual, which leaves the effectiveness of the program among LGBT populations, who have shown greater vulnerability to sexual risk, unaddressed [56]. Finally, we also recognize that there were high attrition rates, a phenomenon usually reported in longitudinal studies [57]. However, these attrition rates did not differ significantly between the groups in the initial phases. Although at the 6-month follow-up phase there were higher attrition rates in the EG, the variables of participant gender, consistent condom use, and percentage of condom use were not related to participant attrition. Future research should evaluate the long-term effectiveness of the program in Colombia, as well as identify the mediating variables of program effectiveness, such as fidelity of implementation, the participants’ individual characteristics [47], and the program facilitators’ individual characteristics [11, 19]. Finally, it is important that the effectiveness of the program be tested in vulnerable populations in different regions of Colombia, perhaps using technological tools to increase program coverage in remote areas. It is hoped that research in these avenues could lead to a decrease in the rate of STIs and unplanned pregnancies among Colombian adolescents.

## Conclusions

This study presents a specific version of the COMPAS that has been adapted culturally to Colombian populations. The program consists of five sessions, validated using a cluster-randomized control trial, and shows high efficacy levels. This version of the COMPAS will be of great use in preventing sexual risk behaviours in adolescents in Colombia.

## Data Availability

Not applicable.

## References

[1] UNAIDS. El sida en cifras. https://www.unaids.org/es. Accessed 8 Aug 2021.

[2] UNICEF. Adolescent HIV prevention: In order to ramp up our efforts in the fight against AIDS, there is a need for more concentrated focus on adolescents and young people. https://data.unicef.org/topic/hivaids/adolescents-young-people/. Accessed 10 Aug 2021.

[3] UNAIDS. People living with HIV receiving art—as of 30 June. https://aidsinfo.unaids.org/. Accessed 8 Oct 2020.

[4] ONUSIDA. HIV and AIDS Estimates, Colombia. https://www.unaids.org/es/regionscountries/countries/colombia. Accessed 8 Oct 2020.

[5] OPS U&U. Acelerar el progreso hacia la reducción del embarazo en la adolescencia en América Latina y el Caribe. ttps://www.unicef.org/lac/media/1341/file/PDF%20Acelerar%20el%20progreso%20hacia%20la%20reducci%C3%B3n%20del%20embarazo%20en%20la%20adolescen.pdf. Accessed 8 Oct 2020.

[6] Profamilia & Plan International. Determinantes del embarazo en adolescentes en Colombia. https://www.minsalud.gov.co/sites/rid/Lists/BibliotecaDigital/RIDE/VS/ED/PSP/informe-determinantes-sociales-embarazo-en-adolescente.pdf. Accessed 8 Oct 2020.

[7] Morales A, Vallejo-Medina P, Abello-Luque D (2018). Sexual risk among Colombian adolescents: knowledge, attitudes, normative beliefs, perceived control, intention, and sexual behavior. BMC Public Health.

[8] Picot J, Shepherd J, Kavanagh J (2012). Behavioural interventions for the prevention of sexually transmitted infections in young people aged 13–19 years: a systematic review. Health Educ Res.

[9] Kaufman MR, Cornish F, Zimmerman RS, Johnson BT (2014). Health behavior change models for HIV prevention and AIDS care: practical recommendations for a multi-level approach. J Acquir Immune Defic Syndr.

[10] Bailey J, Mann S, Wayal S, Abraham C, Murray E. Digital media interventions for sexual health promotion—opportunities and challenges. BMJ. 2015;350. 10.1136/bmj.h1099 25736806

[11] Albarracín D, Durantini MR, Earl A (2006). Empirical and theoretical conclusions of an analysis of outcomes of HIV-prevention interventions. Curr Dir Psychol Sci.

[12] Durantini MR, Albarracin D, Mitchell AL, Earl AN, Gillette JC (2006). Conceptualizing the influence of social agents of behavior change: A meta-analysis of the effectiveness of HIV-prevention interventionists for different groups. Psychol Bull.

[13] Fisher JD, Fisher WA, Shuper PA. The information-motivation-behavioral skills model of HIV preventive behavior. Second Edition ed. New York: Jossey-Bass San Francisco, CA, 2009.

[14] Fisher JD, Fisher WA (1992). Changing AIDS-risk behavior. Psychol Bull.

[15] Bandura A, Rivière Á. Teoría del aprendizaje social. 2nd ed. Madrid, España: Espasa; 1982.

[16] Bandura A. Social cognitive theory of social referencing. In: Feinman S, editor. Social referencing and the social construction of reality in infancy. Second Edition ed. Madrid, España: Springer; 1992.

[17] Fishbein M (2008). A reasoned action approach to health promotion. Med Decis Making.

[18] Ajzen I (2001). Nature and operation of attitudes. Annu Rev Psychol.

[19] Morales A, Espada JP, Orgilés M, Escribano S, Johnson BT, Lightfoot M (2018). Interventions to reduce risk for sexually transmitted infections in adolescents: a meta-analysis of trials, 2008–2016. PLoS ONE.

[20] Morales A, Espada JP, Orgilés M, Secades-Villa R, Remor E (2014). The short-term impact of peers as co-facilitators of an HIV prevention programme for adolescents: A cluster randomised controlled trial. Eur J Contracept Reprod Health Care.

[21] Espada E, Morales O (2016). A two years’ follow-up evaluation of a sexual-health education program for Spanish adolescents compared to a well-established program. Eval Health Prof.

[22] Escribano S, Espada JP, Morales A, Orgilés M (2015). Mediation analysis of an effective sexual health promotion intervention for Spanish adolescents. AIDS Behav.

[23] Espada JP, Orgilés M, Morales A, Ballester R, Huedo-Medina TB (2012). Effectiveness of a school HIV/AIDS prevention program for Spanish adolescents. AIDS Educ Prev.

[24] Morales A, Garcia-Montaño E, Barrios-Ortega C, Niebles-Charris J, Garcia-Roncallo P, Abello-Luque D (2019). Adaptation of an effective school-based sexual health promotion program for youth in Colombia. Soc Sci Med.

[25] Vallejo-Medina P, Correa JC, Gómez-Lugo M (2020). A text mining approach for adapting a school-based sexual health promotion program in Colombia. Prev Med Rep.

[26] Bandura A, Walters RH. Social learning theory. 2nd ed. NJ: Prentice-hall Englewood Cliffs; 1977.

[27] Espada JP, Morales A, Orgilés M, Méndez FX. Programa COMPAS. Competencias para adolescentes con una sexualidad saludable. De la emoción al sentido. 1ª ed. España: Ediciones Pirámide; 2018.

[28] Cheng Y, Lou C, Mueller LM (2008). Effectiveness of a school-based AIDS education program among rural students in HIV high epidemic area of China. J Adolesc Health.

[29] Espada JP, Guillen-Riquelme A, Morales A, Orgiles M, Sierra JC (2014). Validation of an HIV and other sexually transmitted infections knowledge scale in an adolescent population. Aten Primaria.

[30] Abello-Luque D, Espada JP, García-Montaño E, Gómez-Lugo M, Morales A, Pérez-Pedraza D (2021). Colombian adaptation of the HIV and other sexually transmitted infections knowledge scale (KSI) in an adolescent population. Eval Health Prof.

[31] Espada JP, Ballester R, Huedo-Medina T, Secades-Villa R, Orgilés M, Martínez-Lorca M (2013). Development of a new instrument to assess AIDS-related attitudes among Spanish Youngsters. AN PSICOL.

[32] Gómez-Lugo M, Morales A, Saavedra-Roa A (2020). Psychometric properties of the Colombian version of the HIV attitudes scale for adolescents. Int J Environ.

[33] Vallejo-Medina P, Gómez-Lugo M, Marchal-Bertrand L, Saavedra-Roa A, Soler F, Morales A (2017). Desarrollo de guías para adaptar cuestionarios dentro de una misma lengua en otra cultura. Terapia Psicológica.

[34] World Health Organization. Infecciones de Transmisión Sexual (Nota descriptiva N° 10). http://www.who.int/mediacentre/factsheets/fs110/es/. Accessed 8 Oct 2020.

[35] Sanders SA, Yarber WL, Kaufman EL, Crosby RA, Graham CA, Milhausen RR (2012). Condom use errors and problems: a global view. Sex Health.

[36] González-Hernández AM, Escobar-Estupinan JL, Vallejo-Medina P (2020). Condom use errors and problems in a sample of young colombian adults. J Sex Res.

[37] Rosenthal R, Rosnow RL (1985). Contrast analysis: Focused comparisons in the analysis of variance.

[38] Liang K, Zeger SL (1986). Longitudinal data analysis using generalized linear models. Biometrika.

[39] Morales A, Carratalá E, Orgilés M, Espada JP (2017). Un estudio preliminar de la eficacia de un programa de promoción de la salud sexual en adolescentes con padres divorciados. Health Addict /Salud Drog.

[40] Espada JP, Morales A, Orgilés M, Jemmott JB, Jemmott LS (2015). Short-term evaluation of a skill-development sexual education program for Spanish adolescents compared with a well-established program. J Adolesc Health.

[41] Morales A, Espada JP, Orgilés M (2015). A 1-year follow-up evaluation of a sexual-health education program for Spanish adolescents compared with a well-established program. Eur J Public Health.

[42] Fishbein A (2011). Predicting and changing behavior: The reasoned action approach.

[43] Armitage CJ, Conner M (2001). Efficacy of the theory of planned behaviour: a meta-analytic review. Br J Soc Psychol.

[44] Ajzen I (1991). The theory of planned behavior. Organ Behav Hum Decis Process.

[45] Sheeran P, Abraham C, Orbell S (1999). Psychosocial correlates of heterosexual condom use: a meta-analysis. Psychol Bull.

[46] Espada JP, Morales A, Guillén-Riquelme A, Ballester R, Orgilés M (2015). Predicting condom use in adolescents: a test of three socio-cognitive models using a structural equation modeling approach. BMC Public Health.

[47] Escribano S, Espada JP, Orgilés M, Morales A (2016). Implementation fidelity for promoting the effectiveness of an adolescent sexual health program. Eval Program Plann.

[48] DANE. Medida de pobreza multidimensional municipal de fuente censal 2018. https://www.dane.gov.co/index.php/estadisticas-por-tema/pobreza-y-condiciones-de-vida/pobreza-y-desigualdad/medida-de-pobreza-multidimensional-de-fuente-censal. Accessed 13 Oct 2020.

[49] Instituto Nacional de Estadística, INE. Nacimientos 2017. https://www.ine.es/jaxi/Datos.htm?path=/t20/e301/nacim/a2017/&file=02001.px#!tabs-tabla. Accessed 8 Oct 2020.

[50] Dolcini MM, Harper GW, Watson SE, Catania JA, Ellen JM. Friends in the ‘hood: Should peer-based health promotion programs target nonschool friendship networks? J Adolesc Health. 2005;36(3):267. e6–267. e15. 10.1016/j.jadohealth.2004.10.003 15737785

[51] DANE. Nacimientos 2018. https://www.dane.gov.co/index.php/estadisticas-por-tema/salud/nacimientos-y-defunciones/nacimientos/nacimientos-2018. Accessed 13 Oct 2020.

[52] OECD & Ministerio de Educación de Colombia. Revisión de políticas nacionales de educación: La educación en Colombia. Organización para la Cooperación y el Desarrollo Económicos. France: París; 2016.

[53] Arrivillaga M, Correa D, Tovar LM, Zapata H, Varela MT, Hoyos PA (2011). Infecciones de transmisión sexual en la región Pacífica colombiana: implicaciones para población en situación de vulnerabilidad étnica, social y económica. Pensamiento Psicológico.

[54] García-Corzo JR, Tarazona-Álvarez Y, Rojas-Gómez JP, Bayona-Millán EdP, Díaz-Martínez LA (2016). Conocimientos sobre la transmisión del virus de la inmunodeficiencia humana entre estudiantes de 11 a 20 años de comunas pobres de Bucaramanga, Colombia. Arch Argent Pediatr.

[55] Pallitto CC, O’Campo P (2005). Community level effects of gender inequality on intimate partner violence and unintended pregnancy in Colombia: testing the feminist perspective. Soc Sci Med.

[56] Blake SM, Ledsky R, Lehman T, Goodenow C, Sawyer R, Hack T (2001). Preventing sexual risk behaviors among gay, lesbian, and bisexual adolescents: the benefits of gay-sensitive HIV instruction in schools. Am J Public Health.

[57] Malow RM, Kershaw T, Sipsma H, Rosenberg R, Dévieux JG (2007). HIV preventive interventions for adolescents: a look back and ahead. Curr HIV/AIDS Rep.

